# Circular of State Medical Society Respecting Voluntary Medical Associations

**Published:** 1847-03

**Authors:** 


*Circular of State Medical Society respecting Voluntary Medical Associations.*

Dear Sir—The following Preamble and Resolutions, unanimously adopted by the N. Y. State Medical Society at their present session, have been referred to us as the Committee instructed to correspond with the several County Societies, and invite their co-operation in our endeavors to elevate the standing and extend the usefulness of the Medical profession. Permit the Committee, in addition, to express their opinion that in no way can Physicians better promote the great interests of their profession, which are identical with those of the community at large, than by cultivating good feeling among themselves, and a rigid observance of the rules of medical ethics as adopted by our State Society in 1823, a copy of which should be in the hands of every practitioner.

Will you please to call the attention of your Society, or of medical gentleman in your vicinity, to this subject.

THOMAS W. BLATCHFORD,<br>

SN. S. DAVIS, } Committee.<br>

UMNER ELY,

*Albany, Feb.* 4, 1847.

"*Whereas*, Quackery, of all descriptions and grades, physical, and extra physical, crude, sublimated and transcendentalized, with and without substance, ponderous and imponderable, was never more rife in community, and never stood forth with a bolder front, than at the present time ; and whereas, in the minds of many estimable individuals it has become so confounded with the scientific practice of our profession as readily to beguile the unwary, the duty seems imperatively forced upon us, as the accredited guardians of the public health, to disabuse the minds of the people as far as practicable, that they may understand that between quackery and science there is an irreconcilable difference—a bridgeless gulf ; therefore

"*Resolved*, That this society highly approve of the organization in the city of New-York, of the "New-York Academy of Medi-

CINE," and that in the harmony and full attendance which characterize their meetings, (from 100 to 200 being their usual number,) we discover the harbinger of brighter days, the pledge of extetended usefulness.

"*Resolved*, That as far as it may be deemed practicable, it be recommended that voluntary associations of physicians be formed, whose duty it shall be to hold frequent meetings, to read dissertations on medical subjects ; to relate interesting cases of disease ; and interchange views with each other on all subjects connected with our profession ; that thus, interesting and instructive matter may be in readiness to be laid before the respective county societies at their regular meetings, which will tend to call out the latent talent of the profession, and make our meetings something more than mere medical elections, while at the same time it will make manifest before the public, the wide difference between science and quackery."

